# Does External Exposure of Glycidol-Related Chemicals Influence the Forming of the Hemoglobin Adduct, *N*-(2,3-dihydroxypropyl)valine, as a Biomarker of Internal Exposure to Glycidol?

**DOI:** 10.3390/toxics8040119

**Published:** 2020-12-13

**Authors:** Yuko Shimamura, Ryo Inagaki, Hiroshi Honda, Shuichi Masuda

**Affiliations:** 1 School of Food and Nutritional Sciences, University of Shizuoka, 52-1 Yada, Suruga-ku, Shizuoka 422-8526, Japan; shimamura@u-shizuoka-ken.ac.jp (Y.S.); s16601@u-shizuoka-ken.ac.jp (R.I.); 2 KAO Corporation, R&D Safety Science Research, 2606 Akabane, Ichikai-Machi, Haga-Gun, Tochigi 321-3497, Japan; honda.hiroshi@kao.com

**Keywords:** glycidol, glycidyl fatty acid esters, Hb adduct, *N*-(2.3-dihydroxypropyl)valine

## Abstract

Glycidyl fatty acid esters (GE) are constituents of edible oils and fats, and are converted into glycidol, a genotoxic substance, in vivo. *N*-(2,3-dihydroxypropyl)valine (diHOPrVal), a hemoglobin adduct of glycidol, is used as a biomarker of glycidol and GE exposure. However, high background levels of diHOPrVal are not explained by daily dietary exposure to glycidol and GE. In the present study, several glycidol-related chemicals (glycidol, (±)-3-chloro-1,2-propanediol, glycidyl oleate, epichlorohydrin, propylene oxide, 1-bromopropane, allyl alcohol, fructose, and glyceraldehyde) that might be precursors of diHOPrVal, were administered to mice, and diHOPrVal formation from each substance was examined with LC-MS/MS. DiHOPrVal was detected in animals treated with glycidol and glycidyl oleate but not in mice treated with other chemicals (3-MCPD, epichlorohydrin, propylene oxide, 1-bromopropane, allyl alcohol, fructose, and glyceraldehyde). The amount of diHOPrVal per administered dose produced from other chemicals was negligible compared to the amounts associated with dietary glycidol and GE. The present study provides important knowledge for exploring other sources for internal exposure to glycidol.

## 1. Introduction

Glycidyl fatty acid esters (GE) are process contaminants, formed in a deodorization process under high-temperature conditions during the production of edible oils. Diacylglycerol oil was found to contain GE at considerably higher levels than other commercial edible oils. GEs were also formed in heat treatment of saltwater fish, meat patties, etc., especially at high temperature using a charcoal grill [1,2]. Furthermore, the formation of GE can occur in all refined edible oils and processed foods made using these oils [3,4], infant formula [5], baby foods [6], etc.

GE are degraded in vivo by lipase to produce glycidol (2,3-epoxy-1-propanol), a reactive epoxide. Glycidol is a confirmed rodent carcinogen in a National Toxicology Program (NTP) study [7]. The genotoxicity is well characterized in vitro [8,9] and in vivo [10]. Human exposure is a concern because glycidol would be produced from dietary GE.

Hemoglobin adducts are used as biomarkers for evaluating long-term exposure to various reactive chemicals. *N*-(2,3-dihydroxypropyl)valine (diHOPrVal) is a hemoglobin adduct of glycidol and is used as a marker for internal exposure to glycidol [11,12,13,14]. A total of eleven healthy participants consumed a daily portion of about 36 g of commercially available palm fat (8.7 mg glycidol/kg) over 4 weeks. The mean daily glycidol exposure, as estimated from the adduct levels of the participants before the intervention period (background levels of diHOPrVal), was 0.94 µg/kg body weight [15]. Additionally, diHOPrVal levels were used to estimate a continuous exposure of 1.4 μg/kg/day of glycidol in 50 children aged approximately 12 years old [16]. These values are significantly higher than the European Food Safety Authority estimates of intake for adults and children. One possible reason for this discrepancy is exposure to other chemicals that form diHOPrVal.

**Figure 1:**
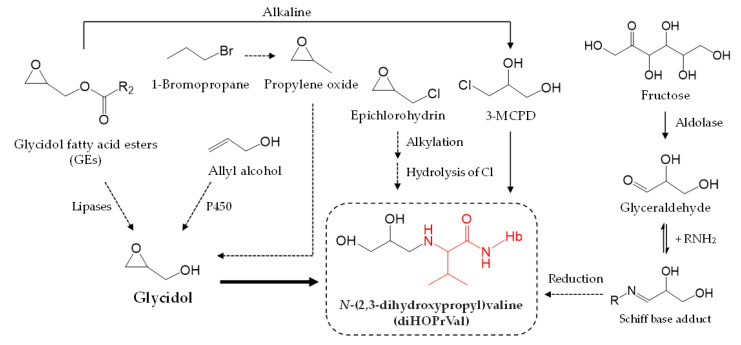
Examples of possible precursors to *N*-(2,3-dihydroxypropyl)valine cf. [16,19].

Theoretically, diHOPrVal might originate from other precursors (Figure 1). One possibility is the food contaminant 3-monochloropropane-1,2-diol (3-MCPD), which often forms in foods in parallel with glycidol [17]. GE are also formed from 3-MCPD esters in neutral, acidic, and alkaline media [18]. Furthermore, allyl alcohol can theoretically be converted to diHOPrVal, and the glyceraldehyde hemoglobin adduct might be converted to diHOPrVal under reducing conditions [19]. Honda et al. reported a positive correlation between intake of one food item (western confectionery) and diHOPrVal levels among the 70 food items and 97 nutrients in a food frequency questionnaire [12], although Aasa et al. did not confirm the importance of sweets intake [16]. Fructose in western confectionery is metabolized to glyceraldehyde [20]. Occupational exposure to epichlorohydrin may also result in diHOPrVal [21]. Ishidao et al. reported glycidol in urine samples of rats that were exposed to 1-bromopropane and assumed that propylene oxide is a possible intermediate between 1-bromopropane and glycidol [22]. diHOPrVal levels in smokers are higher than those in non-smokers [23]. On the other hand, Eckert et al. and Andreoli et al. reported that levels of 2,3-dihydroxypropyl mercapturic acid, a urinary biomarker of glycidol, were correlated with urinary creatinine but not with smoking status [24,25]. It is possible that chemicals other than glycidol might affect the formation of diHOPrVal. Although several studies discussed other possible precursors of glycidol, experimental verification has not been provided. The aim of this study was to examine whether external exposure to glycidol-related chemicals (3-MCPD, epichlorohydrin, propylene oxide, 1-bromopropane, allyl alcohol, fructose, and glyceraldehyde) affects levels of diHOPrVal in vivo using mice. In addition, adducts of epichlorohydrin and glyceraldehyde were evaluated.

## 2. Materials and Methods

### 2.1. Chemicals

Fluorescein isothiocyanate (FITC), glycidol, (±)-3-chloro-1,2-propanediol (3-MCPD) (purity 98.0%), and glycidyl oleate (purity 98.0%) were obtained from Sigma-Aldrich (St Louis, MO). Epichlorohydrin, propylene oxide, 1-bromopropane, allyl alcohol, D(-)-fructose, and DL-glyceraldehyde were obtained from FUJIFILM Wako Pure Chemical Industries Ltd. (Osaka, Japan). L-Valine-(^13^C_5_) (purity 98.0%) used for the synthesis of the internal standard *N*-(2,3-dihydroxypropyl)-(^13^C_5_)valine, were obtained from Cambridge Isotope Laboratories, Inc. (Tewksbury, MA, USA). All other chemicals and solvents used were analytical grade.

### 2.2. Synthesis of Hemoglobin Adducts of Glycidol (diHOPrVal), Epichlorohydrin, and Glyceraldehyde

L-valine (1.17 mg, 10 mmol), sodium hydroxide (0.4 g, 10 mmol), and deionized water (10 mL) were mixed. Glycidol (74 mg), epichlorohydrin (92 mg), and glyceraldehyde (90 mg), respectively, were added to 1 mL of this solution and heated at 60 °C for 18 h. For the internal standard *N*-(2,3-dihydroxypropyl)-(^13^C_5_)valine, L-Valine-(^13^C_5_) (1.17 mg, 10 mmol), sodium hydroxide (0.4 g, 10 mmol), and deionized water (10 mL) were mixed. Glycidol (74 mg) was added to 1 mL of this solution and heated at 60 °C for 18 h. Potassium bicarbonate (0.125 M, 3.0 mL) and 0.5 M FITC in dioxane (2 mL) were added to 1 mL of this solution and heated at 45 °C for 90 min. This mixture was acidified by the addition of 2 mL of 1 N hydrochloric acid. Extraction was conducted with a solution of deionized water:ethyl acetate (1:1, 30 mL). The organic layer was washed twice with deionized water and dehydrated with sodium sulfate. The mixture was evaporated under reduced pressure. The purification used thin-layer chromatography (toluene:ethyl acetate:ethanol = 3:3:1).

### 2.3. Animals and Glycidol-Related Chemicals

Male Institute of Cancer Research (ICR) mice (Japan SLC, Hamamatsu, Japan), 7 weeks old, were used. On arrival, animals were subdivided into controls and test groups (n = 5 per group) such that the mean starting weights were almost identical for all groups. All animals were maintained in a temperature-controlled room (temperature 23 °C ± 1 °C, humidity 55% ± 5%) on a 12 h light/dark cycle and were acclimatized to the laboratory environment for 1 week before the experiment. The dose level and LD_50_ of chemicals was shown in Table 1 [26,27,28,29,30,31,32,33,34]. Glycidol, 3-MCPD, epichlorohydrin, and allyl alcohol with an LD_50_ of 10 mmol/kg bw or less were administered at 0.5 or 1.0 mmol/kg bw for comparison in the same molar dose. GE was administered in equimolar amounts of 0.5 or 1.0 mmol/kg bw for comparison with glycidol. Propylene oxide with an LD_50_ of about 10 was administered at 5.0 mmol/kg bw. Fructose, glyceraldehyde, and 1-bromopropane having an LD_50_ of 10 mmol/kg bw or more were administered at 10 or 18, which is less than half of the LD_50_. They were resuspended in soybean oil, and 8 week-old male ICR mice were treated with single oral doses by gavage, administered 24 h before blood collection. PBS (negative vehicle control) was administered in the same manner. Animals were sacrificed 24 h after the start of treatment, and blood was collected from abdominal vena cava of mice into EDTA and heparin-treated evacuated Venoject tubes (Terumo, Leuven, Belgium). Blood globin (Hb) level was measured with a HemoCue Hb 201+ analyzer (Angelholm, Sweden) and used for derivatization. Animal experimental procedures were conducted with the approval of the Institutional Animal Care and Use Committees of the University (Permit Number: 185182).

### 2.4. Determination of Hemoglobin Adduct by LC-MS/MS

Blood samples were analyzed using the adduct FI*R*E procedure [35]. The principle for this procedure is that adducts on *N*-terminal amino acids are selectively detached and measured after derivatization with isothiocyanate Edman reagents [36]. Whole blood (250 μL) was alkalized with 1 M KHCO_3_ (20 μL), followed by addition of internal standard *N*-(2,3-dihydroxypropyl)-(^13^C_5_)valine. After addition of FITC (5 mg, 13 μmol) dissolved in *N*, *N*-dimethylformamide (30 μL), the sample was heated at 37 °C on a thermomixer comfort for 18 h. Precipitation of proteins by adding acetonitrile (1.4 mL) was performed under slow mixing, followed by centrifugation (10 min at 14,000 × *g*). A pH adjuster (25 μL, 1 M ammonium hydroxide) was added to the supernatant (1.5 mL) before it was transferred to SPE mixed-mode anion exchange cartridges (Oasis MAX, Waters, Milford, MA, USA). A washing procedure with acetonitrile, H_2_O, and 0.5% aqueous cyanoacetic acid (2 mL of each solvent) was performed and the analytes were eluted with 0.25% cyanoacetic acid in acetonitrile (1.4 mL). The solvent was evaporated to dryness under a gentle stream of air and the solid residue was dissolved in 1% formic acid /acetonitrile (80 μL, 1:1, *v*/*v*) prior to analysis. The LC-MS/MS system comprised an HPLC Prominence system (Shimazdu, Kyoto, Japan) and a triple quadruple mass spectrometer, API2000 (AB SCIEX, Tokyo, Japan), equipped with a TurboIonSpray source (electrospray ionization). L-column 2 ODS (2.1 mm diameter, 75 mm length, 5 µm packing materials; Chemical Evaluation and Research Institute, Tokyo, Japan) was used as a separation column. The mobile phase comprised (A) 0.1% formic acid in H_2_O/acetonitrile (4:1, *v*/*v*) and (B) 0.1% formic acid in H_2_O/acetonitrile (1:4, *v*/*v*). A gradient was applied from 0% B to 20% B in 3 min and then stepped to 100% B over 5 min before re-equilibrating the column with the initial mobile phase. The injection volume was 10 µL, and the flow rate was 0.2 mL/min. The column temperature was maintained at 40 °C throughout the separation.

**Figure 2:**
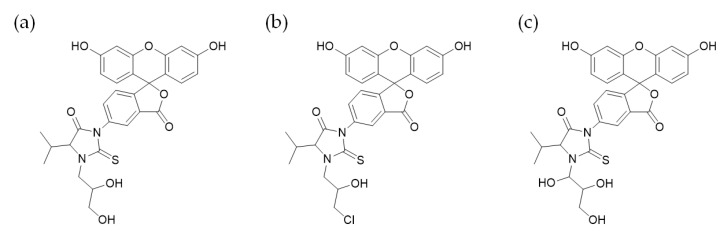
Structures of *N*-terminal valine adduct fluorescein isothiocyanate (FITC) derivative. (**a**) Glycidol-valine adduct FITC derivative, (**b**) epichlorohydrin-valine adduct FITC derivative, and (**c**) glyceraldehyde-valine adduct FITC derivative. An Edman reagent, FITC, is added to L-valine and glycidol, epichlorohydrin, or glyceraldehyde. *N*-terminal valine adducts are derivatized and detached as adduct derivatives, fluorescein thiohydantoins.

Samples were placed in an autosampler at 5 °C. MS conditions were polarity, positive ion mode; curtain gas, 20.0 psi; collision gas thickness, 6; temperature, 500 °C; gas supply 1, 60.0 psi; gas supply 2; 60.0 psi; ion spray voltage, 5500 V. Analysis of samples used positive ion mode with multiple reaction monitoring and the following transitions: comp. glycidol-valine adduct FITC derivative (Figure 2a) *m*/*z* 563→390; epichlorohydrin-valine adduct FITC derivative (Figure 2b) *m*/*z* 581→390; glyceraldehyde-valine adduct FITC derivative (Figure 2c) *m*/*z* 579→390.

The detection limit for the LC-MS/MS was set to a peak height of 3 times the noise. The calibration curve was established as the area ratios between analyte and internal standard versus added amount of analyte per sample. The linearity of the analytical response for each of the analytes was assessed by analysis of the calibration curves samples from ten concentrations in triplicates containing standards. The calibration curve was linear with R’ = 0.999. Analyses in triplicate gave an average coefficient of variation (CV) of 6%.

## 3. Results

**Figure 3:**
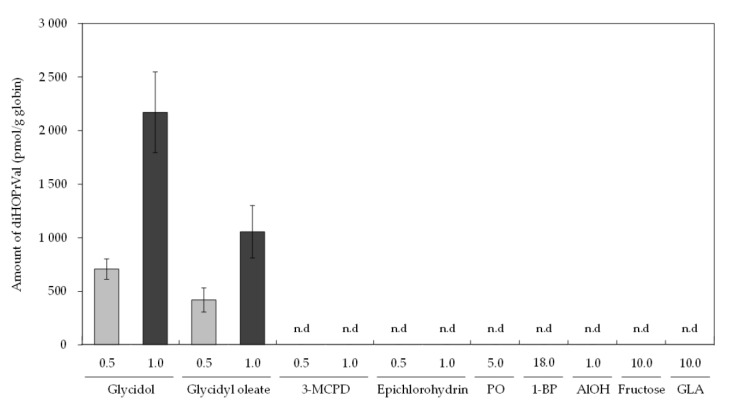
Amounts of diHOPrVal in the blood of mice orally administered glycidol-related chemicals (glycidol, glycidyl oleate, 3-MCPD, and epichlorohydrin, 0.5, 1.0 mmol/kg bw; propylene oxide (PO), 5.0 mmol/kg bw; 1-bromopropane (1-BP), 8.0 mmol/kg bw; allyl alcohol (AlOH), 1.0 mmol/kg bw; fructose, 10 mol/kg bw; glyceraldehyde (GLA), 10 mol/kg bw.

Amounts of diHOPrVal in the blood of mice were measured after the administration of possible precursors to diHOPrVal (Figure 1). Limits of detection (LODs) of diHOPrVal and the specific adducts from epichlorohydrin (Figure 2b), and glyceraldehyde (Figure 2c), respectively, were 10 pmol/g globin. DiHOPrVal was detected after the administration of glycidol (0.5 and 1.0 mmol: 707 ± 43 and 2170 ± 169 pmol/g globin, respectively) and glycidyl oleate (0.5 and 1.0 mmol: 419 ± 50 and 1055 ± 110 pmol/g globin, respectively) (Figure 3). Glycidol and glycidyl oleate induced a concentration-dependent increase in diHOPrVal. However, diHOPrVal was not detected in mice treated with 3-MCPD, epichlorohydrin, propylene oxide, 1-bromopropane, allyl alcohol, fructose, or glyceraldehyde. Furthermore, no detection of the specific epichlorohydrin and glyceraldehyde adducts was observed.

## 4. Discussion

Glycidol-related chemicals considered possible precursors of glycidol diHOPrVal (Figure 1) were administered to mice, and the amount of diHOPrVal in blood was measured. The formation of diHOPrVal was observed only in mice treated with glycidol of glycidyl oleate (GE). The background levels of diHOPrVal typically observed in other studies (5–10 pmol /g globin) [37] was below LOD, probably because of differences in the detection sensitivity of analytical equipment. The present study examined single doses, and an increment in diHOPrVal levels might be detectable after repeated doses. In addition, diHOPrVal in globin of rats after acute exposure of epichlorohydrin (0.11 mmol/kg bw) showed a delayed formation with still high levels 10–20 days following exposure [10]. It is possible that it takes some days to reach detectable diHOPrVal levels in mice after acute exposure. Furthermore, precursor exposure levels are thought to be involved in the formation of diHOPrVal. For example, regarding the exposure level, the recommended hygienic standards in Sweden, based on 8 h time-weighted-average, are set to 0.5 ppm for epichlorohydrin [38]. 2-hydroxypropyl mercapturic acid (2-HPMA, biomarker for propylene oxide) of the median levels for smokers and non-smokers were 49.8 and 11.5 μg/L, respectively [24]. Allyl alcohol in oil-cooked garlic was reported to be 6.1 mg/100 g [39]. From these previous studies, doses of chemicals other than glycidol used in this study were much higher than assumed daily exposure levels. Therefore, internal exposure to glycidol from external exposure to 3-MCPD, epichlorohydrin, propylene oxide, 1-bromopropane, allyl alcohol, fructose, and glyceraldehyde was considered negligible.

Results for detection of diHOPrVal by administration of GE and 3-MCPD in vivo are consistent with previous reports. Appel et al. orally administered equimolar amounts of glycidol or GE into male Wistar rats and examined the biotransformation rate of GE to glycidol. Blood levels of diHOPrVal reached equivalent levels after the administration of either substance [40]. Aasa et al. reported that 3-MCPD had about 1000 times slower reactivity than glycidol towards the *N*-terminal valine in hemoglobin [17]. Therefore, 3-MCPD would make only a small contribution to diHOPrVal formation. Furthermore, El Ramy et al. reported that a dose of 3-MCPD that showed significant DNA damage was approximately 100 times higher than that of glycidol [8]. Hemoglobin adduct formation is only useful as an indicator of genotoxicity if the formation of adducts is correlated with DNA damage. Considering the amount of 3-MCPD in the diet and diminished capacity to form diHOPrVal in vivo, 3-MCPD is unlikely to have a substantial role in diHOPrVal formation.

DiHOPrVal was not detected in mice treated with epichlorohydrin and allyl alcohol in the present study (Figure 3). Allyl alcohol is used in the manufacture of food flavorings and is found in garlic. Allyl alcohol can theoretically be a precursor to diHOPrVal since metabolic conversion to glycidol might occur [19]. Allyl alcohol can theoretically be metabolized to glycidol by CYP [19]; however, such metabolism would be limited in mice, since diHOPrVal level was not elevated in allyl alcohol group. diHOPrVal is also used as a biomarker of epichlorohydrin exposure in workers [21]. However, it is difficult to identify the exposure source of epichlorohydrin because of a relatively high background of diHOPrVal of unknown origin [21]. DiHOPrVal was not detected even in mice treated with propylene oxide and 1-bromopropane, which have similar structures to epichlorohydrin and allyl alcohol, respectively. 1-Bromopropane is reported to be metabolized to glycidol and detected in urine after administration, but quantities are not reported [22]. The interpretation of urinary metabolites is uncertain because of unexplained observations, such as a correlation between the amount of creatinine and glycidol urinary metabolites [19]. Possible exposure of glycidol via propylene oxide and 1-bromopropane in the blood and urine is not well known and would need to be explored.

Glyceraldehyde is another possible precursor to diHOPrVal. This chemical is primarily found in tissues as 3-phosphoglyceraldehyde [41]. Glyceraldehyde is produced by an aldol reaction from fructose. Since glyceraldehyde adducts were not detected in mice treated with fructose or glyceraldehyde (Figure 3), Schiff bases are unlikely to occur after single-dose administration; hence, the reduction of protein adducts from Schiff base needed to produce diHOPrVal could not be confirmed. However, the reduction of protein adducts from Schiff bases is reported in vitro in blood [42]. Long-term exposure to glyceraldehyde might also lead to the formation of Schiff bases. Therefore, further analysis with repeated doses would be informative. Moreover, even when relatively large doses of glycidol-related chemicals (3-MCPD, epichlorohydrin, propylene oxide, 1-bromopropane, allyl alcohol, fructose, and glyceraldehyde) were administered, adducts of epichlorohydrin and glyceraldehyde were not detected. Therefore, the amount of epichlorohydrin and glyceraldehyde adducts produced from these glycidol-related chemicals might be very small.

Chemicals evaluated in the study are theoretical precursors of glycidol, but few reports are available to indicate that glycidol is produced chemically or metabolically from them. Processes may vary among species differences and enzyme activity, but glycidol production from these substances is unlikely to be a primary source of background diHOPrVal.

The present results suggest almost no influence of studied chemicals other than glycidol and GE on the formation of diHOPrVal. The conversion rate of other chemicals to glycidol in vivo and species difference of metabolism are important factors. However, propylene oxide, 1-bromopropane, and allyl alcohol used in this study appear to be converted to glycidol only slowly in mice, and exposure to these glycidol-related chemicals shows limited ability to induce carcinogenicity via glycidol. Carcinogenesis via glycidol production may be associated with low risk. Additionally, diHOPrVal is a useful exposure indicator with high specificity for glycidol and glycidyl chemicals such as GE.

## 5. Conclusions

The amount of diHOPrVal per administered dose produced in vivo from glycidol-related chemicals (3-MCPD, epichlorohydrin, propylene oxide, 1-bromopropane, allyl alcohol, fructose, and glyceraldehyde) was negligible than the amounts associated with dietary glycidol and GE. However, since epichlorohydrin adducts can take some days to reach detectable diHOPrVal levels in vivo, further investigation is needed to clarify the detail. In the future, factors that influence the formation of diHOPrVal in vivo and the sources of glycidol and GE can be identified, and an accurate risk assessment of glycidol and GE in humans can be completed.

## Figures and Tables

**Table 1:** Administered concentrations of glycidol and related chemicals.

Administration Chemical Substance	Concentration (mmol/kg bw)	LD_50_ (mmol/kg bw)	Reference
Glycidol	0.5, 1.0	6.1	[26]
Glycidyl oleate	0.5, 1.0	9.9–10.9	[27]
3-MCPD	0.5, 1.0	1.7	[28]
Epichlorohydrin	0.5, 1.0	2.6	[29]
Propylene oxide	5.0	10.8	[30]
1-Bromopropane	18.0	38.2	[31]
Allyl alcohol	1.0	1.5–1.7	[32]
Fructose	10.0	22.2	[33]
Glyceraldehyde	10.0	33.3	[34]

All LD_50_ values are from oral administration to mice.
